# Anatomic Distribution and Clinical Presentation of Pulmonary Carcinoids: A Single-institutional Study

**DOI:** 10.1016/j.atssr.2024.10.019

**Published:** 2024-11-09

**Authors:** Douglas H. Russ, Julie A. Barta, Nathaniel R. Evans, Gregory C. Kane

**Affiliations:** 1 Division of Medicine, Thomas Jefferson University Hospital, Philadelphia, Pennsylvania; 2 Division of Pulmonary, Allergy, & Critical Care Medicine, Thomas Jefferson University Hospital, Philadelphia, Pennsylvania; 3 Division of Thoracic Surgery, Thomas Jefferson University Hospital, Philadelphia, Pennsylvania

## Abstract

**Background:**

Pulmonary carcinoid tumors (PCTs) have historically manifested predominantly as central lung lesions causing postobstructive symptoms. However, nearly all case series of PCTs to date evaluate cohorts in years before 2010. Because an increased volume of cross-sectional imaging studies results in more frequent detection of incidental lung nodules, we sought to evaluate whether the nature of this cancer’s clinical characteristics may be evolving by studying a contemporary series of patients.

**Methods:**

The study analyzed all patients with a diagnosis of PCT at Thomas Jefferson University Hospital (Philadelphia, PA) between 2006 and 2020. The study evaluated tumor location within the bronchial tree (ie, central or peripheral), the presence or absence of symptoms at diagnosis, patient demographics, and nodule characteristics on computed tomography.

**Results:**

A total of 73 PCTs were analyzed in 72 patients. Most PCTs were detected incidentally (48 of 72; 95% CI: 55%-77%) and located peripherally (41 of 67; 95% CI: 49%-73%). Logistic regression analysis demonstrated that the likelihood of PCTs having these features increased over the study period.

**Conclusions:**

PCTs manifested predominantly as peripheral lung lesions detected incidentally on computed tomography, which contrasts with the existing literature. This may reflect the changing nature of PCT diagnosis as cross-sectional imaging volume has increased. Clinicians should be mindful that incidental solitary pulmonary nodules detected on chest imaging may represent PCT, especially if the lesions are slow growing.

**In Short**

▪ In a series of 72 patients, PCTs manifested predominantly as peripheral lung lesions detected incidentally on CT.
▪ These findings contrast with the existing literature, which evaluated cohorts from earlier decades, and therefore may reflect the changing nature of PCT diagnosis in an era of increasing cross-sectional imaging volumes.
▪ Clinicians should be mindful that incidental SPNs detected on chest imaging may represent PCTs, especially if the lesions are slow growing.

Pulmonary carcinoid tumor (PCT) is a relatively rare neuroendocrine neoplasm accounting for 1% to 5% of all lung cancers.^1^ PCT is subclassified as low-grade (typical) or intermediate-grade (atypical) by the degree of mitotic activity observed on histopathologic examination. Studies demonstrate 10-year overall survival of approximately 90% for typical carcinoid and 50% for atypical carcinoid.^2^ In clinical practice, PCT often manifests as a solitary pulmonary nodule (SPN) on computed tomography (CT) scans, with numerous studies reporting a predominance of centrally located tumors causing postobstructive symptoms on presentation.^2^ Surgical resection remains the standard of care, with systemic chemotherapy playing a limited role in advanced and unresectable disease.^3^

Most studies of PCT to date have analyzed patient cohorts in the decades preceding 2010; data from more recent years is currently limited. With the continued growth of cross-sectional imaging to evaluate disease in the United States and the introduction of lung cancer screening in 2013, the rate of incidental detection of SPNs continues to rise.^4^ We therefore sought to conduct a retrospective study of patients with a diagnosis of PCT at our institution with the aim of characterizing this cancer’s clinical and radiographic features in a more contemporary patient series. We hypothesized that, compared against previous studies, our cohort would demonstrate a higher proportion of PCTs detected as incidental, asymptomatic nodules harboring a peripheral location in the bronchial tree.

## Material and Methods

We performed a retrospective analysis of patients with a diagnosis of PCT at Thomas Jefferson University Hospital (Philadelphia, PA) between 2006 and 2020. Patients were included in the study whether or not they had undergone surgical resection of the tumor at the time of the study. Our institution’s Institutional Review Board granted a waiver for obtaining informed consent for this study.

Patient demographics and symptoms at presentation were analyzed. Tumor pathologic classification was retrieved from biopsy or surgical pathology reports. Tumor anatomic location, size, and radiographic appearance were determined from CT scans retrieved from our institution’s Picture Archiving and Communication System. A single observer took initial nodule measurements, and a second observer with extensive experience managing pulmonary nodules analyzed those nodules that were more difficult to visualize. For tumors without available CT images, lesion size and characteristics were retrieved from radiology reports.

Tumors were classified as central if they were located within segmental bronchi or more proximal airways; tumors involving subsegmental bronchi or more distal airways were labeled peripheral. Tumors were considered symptomatic if the patient experienced cough, wheezing, hemoptysis, dyspnea, chest pain, postobstructive pneumonia, or systemic symptoms deemed by the treating clinician to be caused by the lesion after the pathologic diagnosis of PCT.

All data were analyzed using R Studio software. Logistic regressions were used to assess for relationships between the date of tumor diagnosis and the presence of symptoms or the bronchial location (ie, central or peripheral) within our cohort. Because some patients were missing 1 or several fields of data, we report the effective sample size for each summary statistic.

## Results

Seventy-three PCTs were identified in 72 patients; 1 patient received a diagnosis of 2 synchronous PCTs, which were treated independently for the analysis. Of PCTs with a definitive histological subclassification, most were typical (57 of 70; 81%), and a minority were atypical (13 of 70; 19%). Fifty-nine tumors had been resected at the time of the study. Most tumors were diagnosed between the years 2010 and 2019 (69 of 73; 95%); only 4 were diagnosed before 2010. The frequency of PCTs encountered at our institution increased over the period under study (Supplemental Figure 1).

### Clinical Presentation and Anatomic Distribution

Two-thirds of PCTs were detected incidentally in patients without symptoms attributable to the tumor (48 of 72; 67%, 95% CI, 55%-77%), whereas one-third were detected on chest imaging during diagnostic work-up of tumor-related symptoms (24 of 72; 33%, 95% CI, 23%-45%). Most PCTs were located peripherally (41 of 67; 61%, 95% CI, 49%-73%). PCTs manifesting with symptoms were more likely to be centrally located than asymptomatic tumors (odds ratio, 7.95; 95% CI, 2.5-25.5). The distribution of PCTs by location in the bronchial tree is shown in Table 1.

**Table 1:** Pulmonary Carcinoid Tumor Distribution Within the Bronchial Tree

Site	All Tumors (n =67)^a^	Typical (n = 55)^b^	Atypical (n = 9)	Diagnosed 2010-2014 (n = 22)	Diagnosed 2015-2019 (n = 44)
Central	26 (39)	22 (40)	3 (33)	14 (64)	11 (25)
Mainstem	3	3	0	2	1
Bronchus intermedius	4	3	1	2	1
Lobar	11	10	1	6	5
Segmental	8	6	1	4	4
Peripheral					
Subsegmental-parenchymal	41 (61)	33 (60)	6 (67)	8 (36)	33 (75)

Values are n or n (%).

a The bronchial location of 6 tumors could not be determined because of missing or incomplete radiographic data.

b Three pulmonary carcinoid tumors with an identifiable bronchial location could not be subclassified as typical or atypical on pathologic analysis.

Tumors manifesting symptomatically were diagnosed at an earlier age than incidental tumors (mean difference = −19.8 years; 95% CI, −13.0 years to −26.6 years) (Table 2). The most common presenting symptoms were cough (14 of 24), pneumonia (10 of 24), and chest pain (6 of 24). No PCTs in the cohort presented with carcinoid syndrome.

**Table 2:** Subset Analysis by Tumor Presentation, Subtype, and Diagnosis Year

Variable	All Tumors (n = 73)	Typical (n = 58)	Atypical (n = 13)	Diagnosed 2010-2014 (n = 23)	Diagnosed 2015-2019 (n = 46)	Symptomatic (n = 24)	Asymptomatic (n = 48)
Symptomatic	24 (33)	18 (33)	6 (43)	12 (52)	10 (22)	...	...
Asymptomatic	48 (67)	39 (67)	7 (57)	11 (48)	36 (78)	...	...
Mean age at diagnosis, y	59.6	59.7	59.0	54.5	63.3	46.6	66.4
Mean tumor diameter at first detection, mm	15.7 [8.6] (n = 66)	15.0 [8.1] (n = 54)	19.9 [10.9] (n = 10)	19.4 [11.2] (n = 20)	13.3 [6.0] (n = 43)	18.2 [10.3] (n = 21)	13.5 [6.7] (n = 44)

Values are n (%), mean, or mean [SD].

Logistic regression analysis demonstrated that the odds that a newly diagnosed tumor would be located centrally decreased 24% annually over the study period (95% CI, −7% to −37%) and the odds that a new PCT would manifest symptomatically decreased 16% annually (95% CI, −1% to −29%) (Supplemental Figures 2, 3). PCTs diagnosed in 2010 to 2014 were centrally located at a higher relative frequency than in 2015 to 2019 (64% vs 25%) and were more likely to be symptomatic (52% vs 22%) (Table 1, Table 2).

### Cohort Demographics, Nodule Characteristics, and Surgical Treatment

Summary statistics for patient demographics, tumor size and characteristics, and type of surgical resection are shown in Supplemental Tables 1 and 2.

## Comment

### Clinical Presentation of Pulmonary Carcinoid Tumor

One-third (33%) of tumors in our series were symptomatic at the time of diagnosis, a markedly lower proportion than described in other case series of PCTs in which 55% to 83% of tumors manifested symptomatically.^5^^,^^6^ This finding may be explained by the increasing use of CT imaging in clinical practice, which inherently increases the likelihood that incidental SPNs (a common radiographic presentation of PCT) will be detected. A recent study of several US health systems found that the volume of chest CTs nearly tripled for adults aged 18+ years between 2000 and 2016, with older adults (aged 65+ years) receiving scans at a rate approximately 4 times greater than adults aged <65 years.^7^ In addition, an important 2015 study from the integrated health system Kaiser Permanente demonstrated that the per capita rate of chest CT use increased by 34% and pulmonary nodule identification by 70% between 2006 and 2012.^4^ Meanwhile, all case series we found in the literature included in their cohort patients with diagnoses established in the 1990s or earlier, and only 1 study included patients with diagnoses established after 2010 (Table 3). We were unable to obtain reliable estimates of total cross-sectional imaging volumes at our institution over the study period; however, we suspect that the annual census of CT chest studies has increased in recent years in part because of the rapid growth of our lung cancer screening program, which launched in 2015.^8^ Put together, this cohort may thus be capturing a new clinical landscape of PCT as the volume of cross-sectional studies has ballooned in the 21st century. Should this trend continue, it is possible that the incidental detection of PCT will continue to rise.

**Table 3:** Proportion of Pulmonary Carcinoid Tumors Located in the Peripheral Lung in Selected Studies

First Author	Years Studied	Sample Size, n	Peripheral Location, %	Criteria for Peripheral Classification	Study Limited to Patients Receiving Lung Resection?
Ducrocq^a^	1976-1996	139	27	A	Yes
Garciá-Yuste	1980-2007	304	28	E	Yes
Fink	1980-1999	142	32	B	No
Fiala	1985-2001	96	29	C	Yes
Mezzetti	1980-2001	98	20	E	Yes
Cardillo	1990-2002	163	40	C	Yes
Rea	1968-2005	252	23	C	Yes
Rugge	1993-2003	67	30	A	Yes
Meisinger	1996-2010	28	57	A	No
Aydin	1992-2008	104	23	D	Yes
Zhong	1990-2010	131	21	C	Yes
Yang	1997-2012	44	18	D	Yes
Herde	1989-2009	59	42	B	No
Russ	2006-2020	67^b^	61	A	No

A, Subsegmental bronchus and more distal; B, Segmental bronchus and more distal; C, No visualization on bronchoscopy; D, No visualization on bronchoscopy and lack of atelectasis and postobstructive pneumonia; E, Not specified.

a Evaluated only patients with typical pulmonary carcinoid tumor.

b Number of tumors that could be definitely characterized as central or peripheral.

We suspected that including patients who had not undergone surgical resection could bias our results toward identifying more asymptomatic PCTs. Indeed, of the 14 PCTs in our cohort that were unresected at the time of this study, 12 were asymptomatic at presentation; 5 of these PCTs were detected during work-up for another malignant disease, and 2 went unresected because the patients declined surgical treatment. There was no statistically significant difference in tumor size at the time of diagnosis between unresected and resected tumors (mean difference, 3.5 mm; 95% CI, −3.8 to 10.8 mm); however, the mean age of patients with unresected tumors trended toward being older than those whose tumors were resected (66.2 years vs 58.1 years; mean difference, 8.1 years; 95% CI, −1.1 to 17.3 years; *P* = .08). To assess whether the inclusion of these unresected cases biased our results, we conducted a subset analysis limited to surgically resected tumors (n = 59) and found that symptomatic tumors still constituted a minority of cases (23 of 59; 39%) at a proportion still lower than reported in the literature. Therefore, the inclusion of unresected PCTs does not alone account for the high proportion of asymptomatic PCTs observed in our series.

### Anatomic Distribution of Pulmonary Carcinoid Tumor

Most carcinoids in this series were located peripherally (61%), a finding that contrasts with the available literature (Table 3), in which only 18% to 42% of tumors have been classified as peripheral (with the exception of a small 2011 study of 28 patients in which 57% of tumors were peripheral). Comparison among studies is difficult because no consensus on the definition of central and peripheral lesions exists (Table 3). Despite our classification of central lesions as those involving segmental bronchi or more proximal airways, which biases our study toward classifying more lesions as central when compared with studies that instead label segmental lesions as peripheral, most PCTs in our series were still located peripherally. If we had instead labeled tumors located in segmental airways as peripheral, as do several other studies, then 73% (49 of 67) of the carcinoids in our series could be considered peripheral (Table 1). Because peripheral lesions are more likely to be asymptomatic (see Results), it is plausible that this finding also reflects the increased volumes of CT chest imaging in recent decades, as discussed.

### Intracohort Analysis

Logistic regression analysis demonstrated that the odds of a PCT manifesting symptomatically or having a central location decreased over study period; the trend was particularly strong for tumor location (Supplemental Figures 2, 3). Although the generalizability of this finding is limited by the single-institution design of this study, these trends are consistent with the hypothesis that PCTs are increasingly manifesting as asymptomatic, peripheral nodules in an era of rising CT study volumes.

### Study Limitations

This is a single-institution study, which limits generalizability to other populations. As a retrospective series, many cases were missing 1 or more fields of data, thus increasing susceptibility to bias. In particular, only 54 of 74 PCTs had CT scans available for direct analysis of the lesion; the remainder could be evaluated only through radiology reports. Although this cohort is larger than that of several similar studies (Table 3), it is still relatively small, thereby decreasing the degree of confidence in our estimates.

### Conclusion

We analyzed 73 PCTs diagnosed at our center between 2006 and 2020. In contrast to the existing literature, the majority of PCTs in our cohort manifested incidentally on CT imaging and harbored a peripheral bronchial location. PCTs were more likely to manifest asymptomatically and with a peripheral lung location during later years of the study period. These findings may be attributable to growing volumes of CT studies in recent decades that result in more frequent incidental detection of PCTs as patients accumulate CT studies of the thorax in the later decades of life. Given that cross-sectional imaging use is positioned to grow further in coming years, we anticipate that the detection and subsequent diagnosis of smaller, peripheral, and asymptomatic PCTs in clinical practice will rise further. Clinicians should therefore be mindful that SPNs detected incidentally with smooth or lobulated borders that are slow growing may represent PCT, an indolent yet still malignant cancer.^9^
