# Supplementary figures and images for: Anatomic Distribution and Clinical Presentation of Pulmonary Carcinoids: A Single-institutional Study
Source: Ann Thorac Surg Short Rep. 2024 Nov 9;3(2):299–303. doi: 10.1016/j.atssr.2024.10.019 (PMC12167561; doi:10.1016/j.atssr.2024.10.019)

**Supplemental Figure 1**


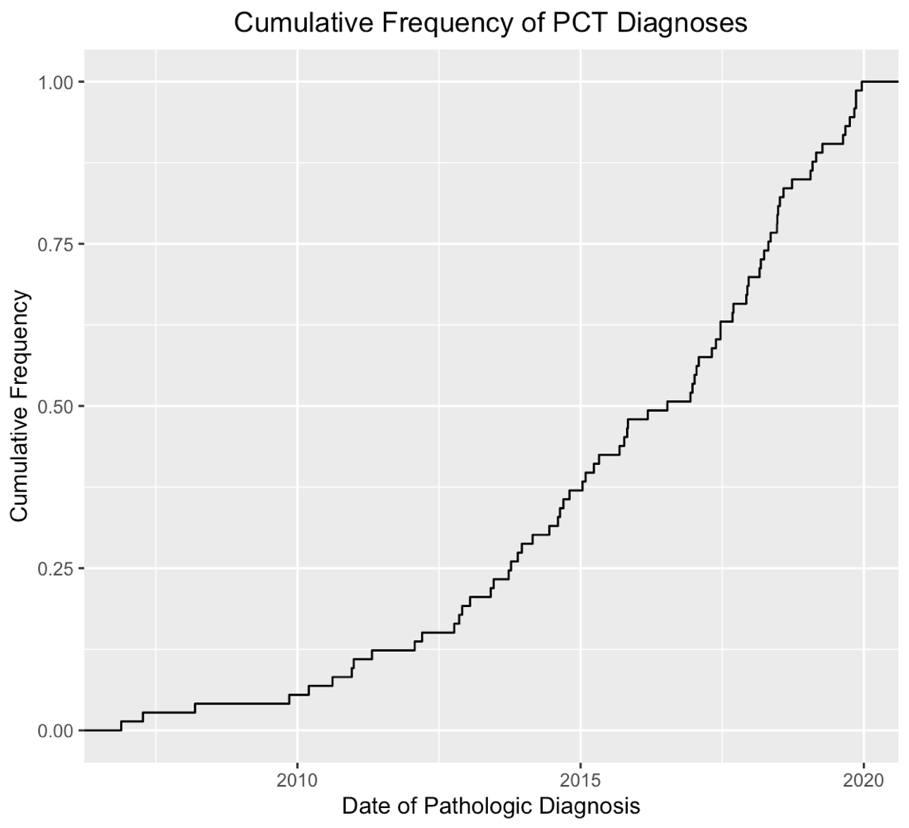

Supplement: Supplementary Figure 1 [file mmc1.docx]

**Supplemental Figure 2**


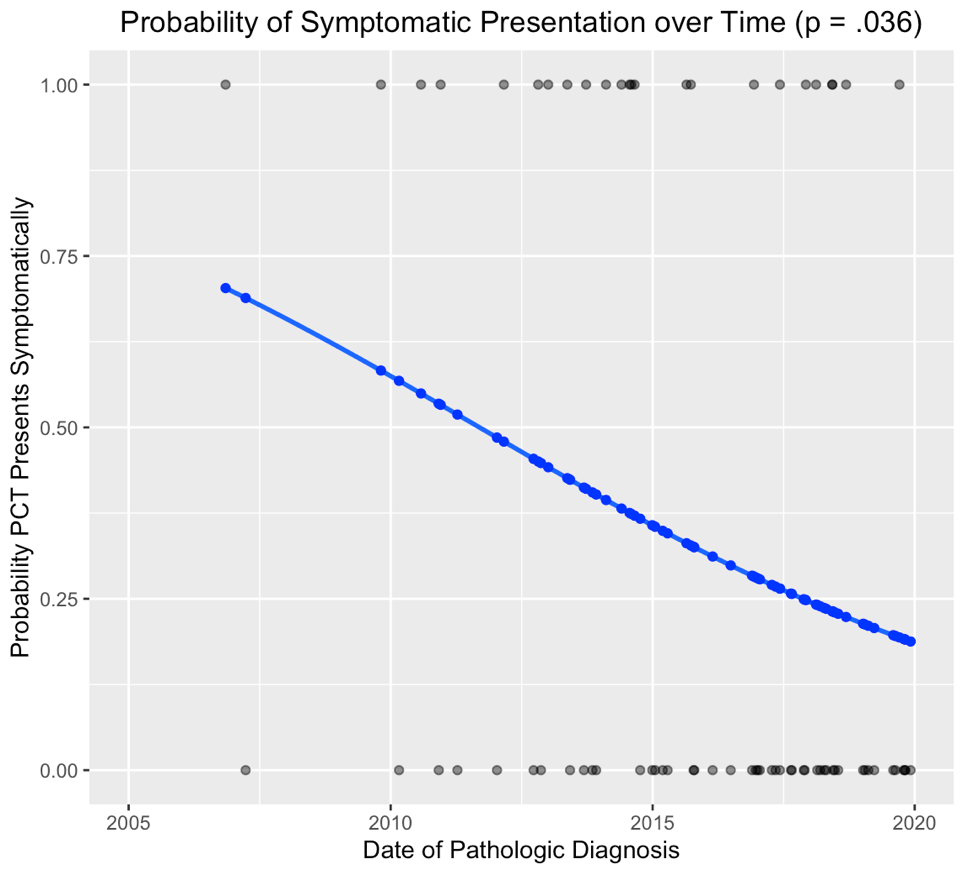

Supplement: Supplementary Figure 2 [file mmc2.docx]

**Supplemental Figure 3**


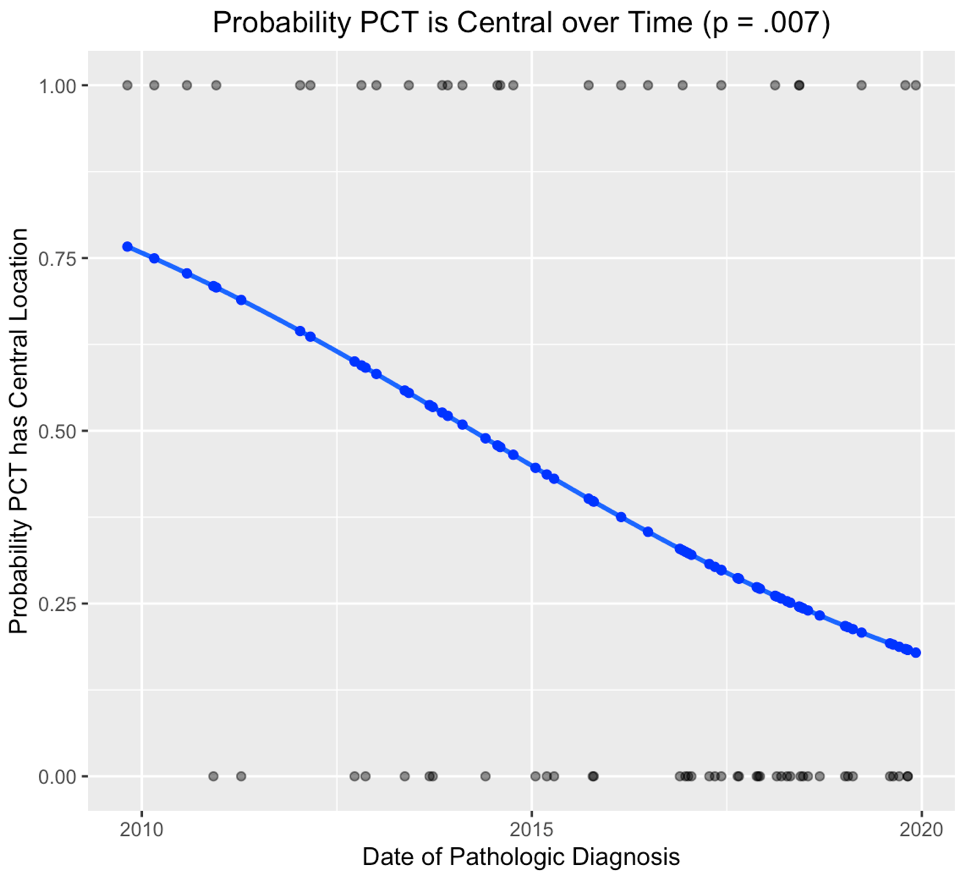

Supplement: Supplementary Figure 3 [file mmc3.docx]
