# Supplementary material for: Anatomic Distribution and Clinical Presentation of Pulmonary Carcinoids: A Single-institutional Study
Source: Ann Thorac Surg Short Rep. 2024 Nov 9;3(2):299–303. doi: 10.1016/j.atssr.2024.10.019 (PMC12167561; doi:10.1016/j.atssr.2024.10.019)
Supplement: Supplementary Table 1 [file mmc4.docx]

**Supplemental Table 1 – Clinical characteristics of PCT by tumor subtype**

|  | All | Typical Carcinoid | Atypical Carcinoid |
| --- | --- | --- | --- |
| Overall Survival |  |  |  |
| 5-year | 84% | 85% | 79% |
| 10-year | 78% | 73% | 79% |
| 15-year | 52% | 73% | 53% |
| Resection Procedure (n=59)^a^ |  |  |  |
| Pneumonectomy | 2 |  |  |
| Lobectomy | 38 |  |  |
| Sleeve Lobectomy | 6 |  |  |
| Segmentectomy | 8 |  |  |
| Wedge resection | 5 |  |  |
| Lung Location (n=73)^b^ |  |  |  |
| RUL | 12 | 9 | 3 |
| RML | 13 | 11 | 1 |
| RLL | 21 | 15 | 6 |
| LUL | 15 | 13 | 1 |
| LLL | 11 | 8 | 3 |
| Nodule Shape (n=52) |  |  |  |
| Smooth or lobulated | 51 |  |  |
| Bilobed | 1 |  |  |
| Nodule Border (n=53) |  |  |  |
| Round or smooth | 52 |  |  |
| Spiculated | 1 |  |  |
| Locoregional Lymph Node Involvement (n=52)^c^ | 4 | 2 | 2 |
| Distant Metastasis | 2 | 1 | 1 |

^a^ 14 biopsy-proven PCTs had not undergone resection at the time of the study

^b^ One endobronchial PCT was located between the distal trachea and proximal right main bronchus and therefore not localized to a lung lobe

^c^ Lymph node status could not be assessed or was unavailable for 7 of 59 resected tumors
