# Supplementary material for: Anatomic Distribution and Clinical Presentation of Pulmonary Carcinoids: A Single-institutional Study
Source: Ann Thorac Surg Short Rep. 2024 Nov 9;3(2):299–303. doi: 10.1016/j.atssr.2024.10.019 (PMC12167561; doi:10.1016/j.atssr.2024.10.019)
Supplement: Supplementary Table 2 [file mmc5.docx]

**Supplemental Table 2 – Expanded subset analysis by tumor clinical presentation, histologic subtype, and year of diagnosis**

|  | All cases (n=73) | Symptomatic (n=24) | Asymptomatic (n=48) | Typical (n=58) | Atypical (n=13) | Diagnosed 2010-2014 (n=23) | Diagnosed 2015-2019  (n=46) |
| --- | --- | --- | --- | --- | --- | --- | --- |
| No. Female Sex (M:F) | 54 (0.35:1) | 19 (0.26:1) | 34 (0.41:1) | 42 (0.38:1) | 10 (0.30:1) | 17 (0.35:1) | 33 (0.39:1) |
| Age at Diagnosis, mean, y | 59.6 | 46.6 | 66.4 | 59.7 | 59.0 | 54.5 | 63.3 |
| No. with smoking history^a^ | 46 (64%)  *(n=72)* | 13 (56%) | 32 (68%)  *(n=47)* | 33 (58%)  *(n=57)* | 11 (82%) | 11 (48%) | 32 (71%) *(n=45)* |
| Tumor Diameter at first detection^b^, mean (SD), mm | 15.7 (8.6) *(n=66)* | 18.2 (10.3) *(n=21)* | 13.5 (6.7) *(n=44)* | 15.0 (8.1) *(n=54)* | 19.9 (10.9) *(n=10)* | 19.4 (11.2) *(n=20)* | 13.3 (6.0) *(n=43)* |
| No. symptomatic^c^ (%) | 24 (33%) | NA | NA | 18 (33%) | 6 (43%) | 12 (52%) | 10 (22%) |
| No. asymptomatic (%) | 48 (67%) | NA | NA | 39 (67%) | 7 (57%) | 11 (48%) | 36 (78%) |

**^a^** Presence or absence of smoking history could not be determined in 1 case

^b^ Tumor diameter at the time of diagnosis could not be determined in 7 cases

^c^ Presence or absence of presenting symptoms could not be determined in 1 case
